# CT guided autologous blood localization of pulmonary ground glass nodules for video assisted thoracoscopic surgery compared to micro-coil localization

**DOI:** 10.1186/s13019-022-01934-3

**Published:** 2022-08-18

**Authors:** Jianxin Xu, Tingting Si, Maohua Zheng, Jun Guan, Zhixin Li, Zhiyang Xu

**Affiliations:** 1 Department of Thoracic Surgery, The Third Clinical College of Fujian Medical University, The First Hospital of Putian, No. 449 Nanmenxi Road, Putian, 351100 China; 2 Department of Thoracic Surgery, Shanghai Pulmonary Hospital, Tongji University School of Medicine, No. 507 Zhengmin Road, Shanghai, 200433 China; 3 Department of Thoracic Surgery, The Affiliated Mindong Hospital of Fujian Medical University, No. 89, Heshan Street, Ningde, Fujian China

**Keywords:** Autologous blood, Micro-coils, Ground-glass nodule, CT-guided localization, Video-assisted thoracoscopic surgery

## Abstract

**Objectives:**

To investigate feasibility and safety of autologous blood in preoperative computed tomography (CT)-guided localization of pulmonary ground-glass nodules (GGNs) by comparing to mico-coil prior to video-assisted thoracoscopic surgery.

**Methods:**

Clinical data of patients with GGNs who underwent video-assisted thoracoscopic surgery followed by preoperative CT-guided autologous blood or micro-coil localization was retrospectively reviewed in our department between September 2019 and November 2021. The localization duration, localization success rate, localization-related complication, localization cost, operation time, and conversion rate were compared between the 2 localization groups.

**Results:**

Totally 65 patients with 65 GGNs were included in our study, with 34 patients in autologous blood group (group B) and 31 patients in micro-coil group (group M). There is no conversion to thoracotomy. The age, sex, nodule location, diameter of nodule and distance from the pleura between the 2 groups were statistically comparable. Compared with group M, group B had similar localization success rate (94.1% vs 83.9%, *P* = 0.183) but shorter localization time (14.50 ± 2.61 min vs 16.35 ± 2.30 min, *P* = 0.004), lower cost ($92.4 ± 3.2 vs $475.6 ± 8.5, *P* = 0.001), and lower incidence of puncture complications (3.0% vs 19.3%, *P* = 0.042).

**Conclusions:**

The autologous blood localization is an effective and more economical method for preoperative GGNs localization, and is associated with fewer complications compared to micro-coil localization.

## Introduction

With the widespread use of lowdose spiral CT and the popularity of lung cancer screening program, an increasing number of pulmonary ground-glass nodules (GGNs) have been detected [1]. Video-assisted thoracoscopic surgery (VATS) provides a minimally invasive surgical resection for diagnosis and treatment of GGNs, which is of high effectiveness and less morbidity compared to traditional thoracotomy. Complete resection by VATS has now become a prevailing therapeutic modality for potential malignant GGNs [2]. However, surgical removal of small pulmonary nodules, especially nonvisible and nonpalpable GGNs, is always challenging under thoracoscopic circumstances. Preoperative nodule localization is therefore of paramount importance for precise resection, which could improve resection accuracy, decrease morbidity, operative time, and also conversion rate to open thoracotomy [3].

Various preoperative localization techniques have been reported includig: hook wire, micro-coil, intraoperative ultrasound, methylene blue, and radio-guided detection etc. Each technique has its own advantages and disadvantages [4]. Computed tomography (CT)-guided autologous blood localization is first introduced in our department from 2019. Our previous experience and some few reports have found it to be an easily obtained and also effective technique for preoperative GGNs localization [5, 6]. However, few comparisons have been made between this novel technique and other localization methods. In this study, we aim to investigate the feasibility of this novel technique in the respect of nodule identification effectiveness and safeties by comparing to CT-guided micro-coils localization.

## Materials and methods

### Patients

Between September 2019 and November 2021, patients with GGNs who underwent preoperative CT-guided localization and subsequent thoracoscopic resection were retrospectively reviewed in our department. The necessity and feasibility of preoperative localization was confirmed by thoracic surgeons and interventional radiologists before the localization procedure. Criteria for localization included: (1) the presence of pulmonary ground-glass nodule in CT scan; (2) less than 20 mm in maximal diameter; (3) located within 2 cm of a pleural surface or fissure; (4) deemed amenable to thoracoscopic wedge excision; (5) with a path for percutaneous puncture. Exclusion criteria for CT-guided localization were the following: (1) GGN located near the bronchi; (2) nodules adjacent to the hilum or apparent vascular structure; (3) patient with surgical contraindications or previous history of thoracic surgery. This study was approved by the review board of The First Hospital of Putian, and the requirement for informed consent for the use of patients’ medical record was waived. All methods were performed in accordance with the Declaration of Helsinki.

### Localization procedure

Patients were positioned in the 64-row multi-slice spiral CT scanner (GE Healthcare, USA). An initial CT scan was obtained to determine the optimal insertion pathway to reach the targeted nodule. All the CT scans and introducer needle insertions were performed during a normal end-inspiration breath hold. Localization procedures were performed on the afternoon before surgery day. All localization procedures were conducted by the same interventional radiologist. Localization duration was defined as from the first CT scan to the last CT scan. Successful targeting was defined as the detection of the marker on the visceral pleura for the target nodule.

### CT-guided autologous blood localization

**Fig. 1:**
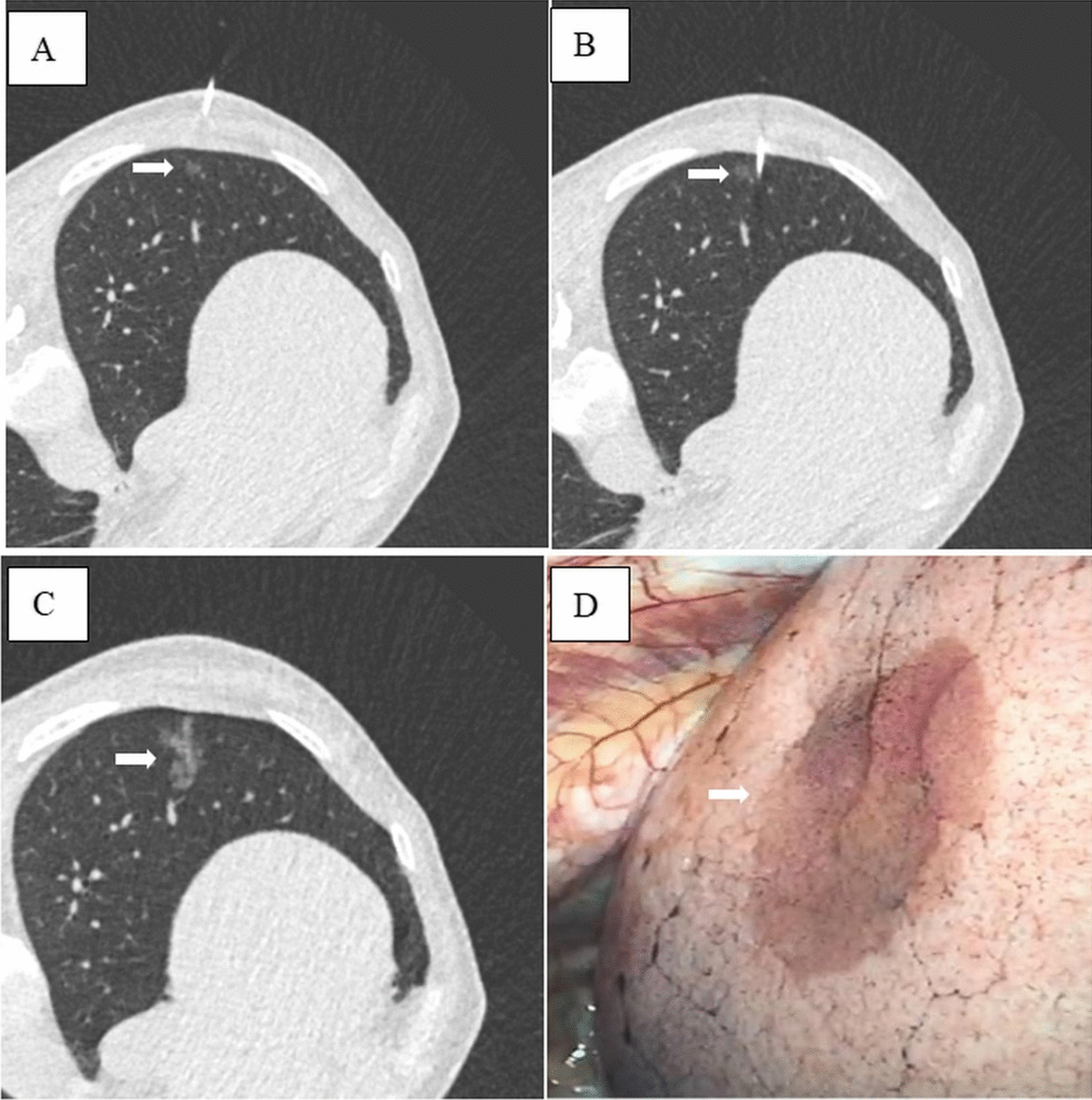
Computed tomography (CT)-guided autologous blood localization on a 65-year-old female with a 8 mm pure ground-glass nodule in the left lower lobe. **A** CT image showing a small pulmonary nodule (arrow) in the left lower lung lobe; **B** CT image showing the coaxial needle placement 5–10 mm caudal to the nodule (arrow); **C** CT image showing the change after the injection of the suspension of autologous blood (arrow); **D** hematoma on the pleura (white arrow) during video-assisted thoracoscopic surgery

In the autologous blood localization group, about 5 mL of blood was drew into a EDTA anticoagulation tube from the patient and the blood tube was then stored in refrigerator under the temperature of 0–4 °C in the ward. During the localization procedure, 2 mL of autologous blood was drew from the anticoagulation tube by the syringe for the localization injection. After local anesthesia, a 20-gauge introducer needle (CareFusion Cooperation) was advanced into the lung parenchyma at least 5 mm beneath the visceral pleura. The stylet was removed after CT confirmation that the tip was in an appropriate position (5–10 mm from the boundary of the target nodule). Thereafter, approximate 0.5–1 mL of the autologous blood was injected. The introducer needle was then gradually withdrawn, autologous blood was injected into the lung parenchyma at the same time. The mean injection dose of autologous blood corresponding to the distance between nodules and the visceral pleura was classified as follows: 0.5 ml autologous blood was injected for a distance < 1 cm, 1 ml autologous blood was injected for a distance between 1 and 2 cm. Finally, the puncture needle was pulled out from chest wall. A postprocedural CT scan was performed to confirm the final location of the autologous blood as well as the punctural complications (Fig. 1). The patient was then sent back to the general ward.

### CT-guided micro-coils localization

**Fig. 2:**
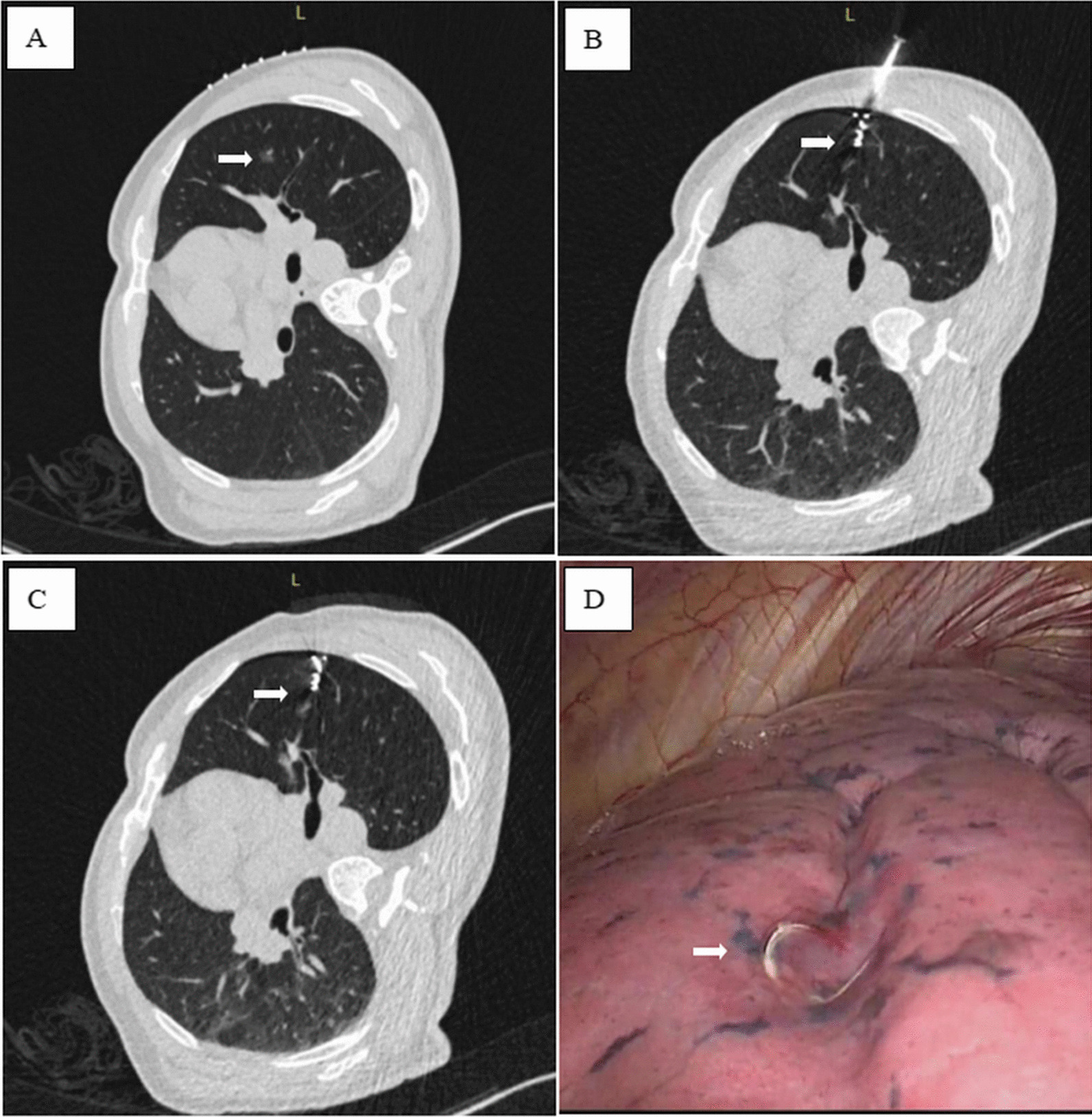
Computed tomography (CT)-guided micro-coil localization on a 48-year-old male with adenocarcinoma in situ in the right upper lobe. **A** CT image showing the lesion (arrow) in the right upper lung lobe; **B** CT image showing introducer needle was inserted into the lung and positioned next to the lesion (arrow); **C** The microcoil was released with the superficial end of the microcoil beyond the visceral pleura and the deep end coiled in the lung parenchyma adjacent to the lesion (arrow). **D** Superficial end of the microcoil (arrow) was visualized during video-assisted thoracoscopic surgery

After determining the appropriate puncture path, local anesthesia was performed with lidocaine. After local anesthesia, a 20G puncture needle was advanced into the lung parenchyma with the needle tip was positioning around the target ground glass nodule. A micro-coil (Cook Company, USA, platinum material, 20G) was then inserted through the introducer needle. The head end of coil is released to the tissue around the targeted nodule, and the tail end is released outside the visceral pleura. As the autologous blood localization did, CT scan was performed to identify the location of the micro-coil and related complications (Fig. 2). The patient was then transferred back to the general ward before surgical procedure.

### Surgical procedure

All patients underwent a one-lung ventilation anesthesia with a double lumen endotracheal tube and then was positioned in the lateral decubitus position. A single incision (about 3 cm) was made through the 4/5th intercostal space in uni-portal VATS. While in multi-portal VATS, the observation port and operation port were selected in 7th and 4th intercostal space or 8th and 5th intercostal space respectively. All VATS procedures were performed under non-direct view without intercostal rib spreading. The location of the nodule was first confirmed by visualizing the superficial end of the microcoil or blood tattoo beyond the visceral pleura. After confirmation of the target lesion site, wedge resection with an adequate margin was performed firstly. The specimen was immediately sent for frozen section to assess whether the lesion was completely resected and whether the surgical margin was enough. Additional anatomic lobectomy and mediastinal lymph node dissection were performed if a nodule was diagnosed as invasive lung cancer with a margin of less than 20 mm. Conversion was defined as conversion to open thoracotomy due to failure to locate the nodule. A 24- or 28-F drainage tube was routinely placed after surgery. Duration for surgical procedure is defined as the time interval between the entrance into the pleural cavity and the completion of a wedge resection in our study. The technical success of the wedge resection was defined when the targeted GGN was found in the resected wedge tissue.

### Statistical analysis

Clinical data, imaging data, localization related data, surgical data and postoperative pathological result were collected from the electronic medical record. Complications were confirmed by postprocedural CT. Statistical analyses were performed using SPSS 25.0 (IBM SPSS statistics, IBM Corp). Continuous variables are presented as means ± standard deviation or medians (range), and categorical variables are presented as counts or rates. A **χ**^2^test or Fisher exact test was used to compare dichotomous variables. A *P*-value less than 0.05 was considered statistically significant.

## Results

From September 2019 to November 2021, preoperative CT-guided localizations by autologous blood or micro-coil were performed for 109 patients with GGNs in our department. Of them, 44 patients were excluded because of simultaneous localization of multiple GGNs. A total of 65 patients with 65 GGNs were finally included in our study. As a result, 34 patients with 34 GGNs in the autologous blood localization group (group B), and 31 patients with 31 GGNs in the micro-coil localization group (group M) were included. In the micro-coil group, targeted lesions consisted of 20 pure GGNs, and 11 part-solid nodules. In the group B, 24 lesions were pure GGNs, and 10 lesions belonged to part-solid nodules. In our study cohort, there were 33 male and 32 females, with age between 38 and 76 years old (55.9 ± 9.8 years old). There was no significant difference between the two groups in terms of gender, age, diameter of nodule, nodule location, and distance of the nodule from the pleura. The characteristics of patients and nodules were listed in Table 1.

**Table 1:** Patient and nodule characteristics

Variables	Group M	GroupB	*P*
Age (y)	56.9 ± 9.3	55.2 ± 10.3	0.489
Gender			0.897
Male	16	17	
Female	15	17	
Diameter of nodule (mm)	12.65 ± 4.46	12.59 ± 4.39	0.959
Distance from the pleura (mm)	16.71 ± 3.77	16.82 ± 3.75	0.903
GGNs Imaging			0.601
Pure GGNs	20	24	
Mixed GGNs	11	10	
Nodule Location			0.726
LUL	10	6	
LLL	4	5	
RUL	7	11	
RML	4	5	
RLL	6	7	

*LUL* left upper lobe, *LLL* left lower lobe, *RUL* right upper lobe, *RML* right middle lobe, *RLL* right lower lobe, *GGNs* ground-glass nodules

The localization success rate was comparable in the localization group, with 83.8% (26/31) in the group M and 94.1% (32/34) in the group B, which showed no significant difference (*P* = 0.183). In the 5 failed cases of group M, the micro-coils were in displacement from the targeted lesions in 2 patients. No nodule was found after a wedge resection in these 2 patients, so segmental resection was then performed. The distal end of the micro-coil had dislodged, and was fixed in thoracic cavity in another 3 cases. Of which, 2 lesions underwent wedge resection after intraoperative nodule identification guided by the small hematoma caused by the puncture, and 1 lesion underwent lobectomy. In the group B, the localization was successful in 32 cases (94.1%) and failed in 2 cases. In the 2 failed cases, there is no distinct hematoma or blood tatoo on the visceral pleural surface after autologous blood injecting due to an extensive anthracotic pigmentation and adhesion. In these 2 patients, 1 patient underwent lobectomy and another patients underwent segmentectomy. The average time of the localization procedure in the group M (16.35 ± 2.30 min) was significantly (*P* = 0.004) longer than that in group B (14.50 ± 2.61 min). For localization procedure costs (in dollars), the microcoil localization costs much more than the autologous blood group ($475.6 ± 8.5 vs $92.4 ± 3.2, *P* = 0.001), which was statistically significant different.

In terms of punctural complications, minor pneumothorax was observed in 1 of 33 (3.1%) patients of the group B. While, in the group M, minor pneumothorax was observed in 4 of 31 (12.9%) patients. Besieds, 1 patient suffered minor haemothorax (3.2%) and 1 patient suffered hemoptysis (3.2%). All pneumothorax in both groups remained asymptomatic and required no additional intervention. Patients with haemothorax and hemoptysis showed mild symptoms and requireed no additional intervention either. We did not observe any pulmonary aeroembolism in both groups. As a result, a overall complication rate was 19.3% in group M and 3.1% in group B, which shows statistically significant difference (*P* = 0.042) (See in Table 2).

**Table 2:** Procedural success rate, cost, complications, and duration of localization for the two groups

Variables	Group M	GroupB	P
Localization success rate	26/31	32/34	0.183
Complication	7/31	1/34	0.042
Pneumothorax	4	1	
Haemothorax	1	0	
Aeroembolism	0	0	
Hemoptysis	1	0	
Duration for localization procedure (min)	16.3 ± 2.7	14.6 ± 2.2	0.007
Cost of localization procedure ($, Dollar)	475.6 ± 8.5	92.4 ± 3.2	0.001

All lesions were successfully removed by VATS without any conversion to thoracotomy, including 57 cases underwent wedge resection, 2 cases underwent segmentectomy, and 6 cases underwent lobectomy. The technical success rate of the VATS-guided wedge resection was respectively 90.3% in group M and 85.3% in group B. (*P* = 0.538)0.2 patients in group M underwent segmentectomy due to a technical localization failure (micro-coil displacement beyond the targeted nodule). In the group B, 1 patient underwent lobectomy due to extensive adhesion and another 1 patient underwent anatomical segmentectomy because of no distinct hematoma or blood tatoo. Four patients underwent additional lobectomy after wedge resection due to the pathological diagnosis of invasive adenocarcinoma. The final pathological result of the targeted lesions was in the agreement with the results of frozen sections (See in Table 3).

**Table 3:** Surgical procedure and pathology of lesions in the two groups

Variables	Group M	GroupB	P
Conversion rate (%)	0	0	
Technical success of wedge resection	90.3%	85.3%	0.538
Surgical procedure			0.103
Wedge resection	28	29	
Segmentectomy/lobectomy	3	5	
Duration for surgical wedge resection (min)	16.9 ± 3.7	22.6 ± 3.3	0.001
Pathology			0.452
Leiomyoma	2 (6.5)	0 (0.0)	
Inflammatory pseudotumor	2 (6.5)	1 (3.0)	
Hamartoma	2 (6.5)	0 (0.0)	
Atypical hyperplasia	1 (3.2)	2 (6.1)	
Adenocarcinoma in situ	13 (41.8)	15 (45.4)	
Minimally invasive adenocarcinoma	7 (22.6)	9 (27.3)	
Invasive adenocarcinoma	4 (12.9)	7 (21.2)	

## Discussion

As one of the most common cancers, lung cancer is the leading cause of cancer death worldwide. With the widespread use of low-dose spiral CT, more and more pulmonary nodules, especially ground glass nodules (GGNs) have been detected. Currently, the diagnosis of pulmonary nodules mainly relied on imaging features of CT scan, positron emission tomography-CT (PET-CT), needle biopsy or bronchoscopy, but the diagnostic accuracy was still not satisfied. The need for accurate early diagnosis of these asymptomatic small GGN lesions with high suspicion of malignancy is vital. For those highly suspected malignant GGNs, VATS has become a preferred diagnostic and treatment option due to its minimal invasiveness and effective pathological confirmation. But it is still challenging for surgeons to resect these small non-visible and non-palpable GGNs without preoperative localization, especially when the nodule is < 10 mm in size, and is > 5 mm from the pleural surface. The failure rate of nodular detection is reported as high as approximately 63% [7]. Preoperative nodule localization is therefore of paramount necessity for a precise VATS resection.

Various preoperative localization techniques (ie, hook wire, microcoil, lipiodol mixture, and methylene blue etc.) has been developed to facilitate the intraoperative identification by thoracic surgeons during VATS. But there is still a controversy on the most efficient and safty one [8]. One of the most commonly applied is the hook-wire techniques. Although this method demonstrated to be effective, the main issues are its high dislodgment and complication rate [9, 10]. Besides, the stiff and sharp hookwire caused torsional stress in lung parenchyma and movement in chest wall, which result in much discomfort or pain. Therefore, the interval time between hookwire localization and VATS should be as short as possible, which is adverse to a casual surgical schedule [11]. The metal coils are not related to the chest wall and there is no movement risk during transfer, which should reduce the risk of migration and complication [12, 13]. Although the placement of microcoils is a relatively feasible method, it also presents a risk of radiation exposure and coil migration [14–16]. Additionally, a microcoil placement may incur an increased risk of gas embolism [17]. Instead of metal implants, other studies also try to employ liquid material (lipiodol [18], methylene blue [19, 20] etc) as the marking dye for nodule localization. However, methylene blue diffuses into surrounding tissue quickly, which can confound and complicate the local surgical resection. Being unnatural chemical industrial material, lipiodol always causes allergies, irritable cough and even vascular embolism during localization process. As an easily absorbable and natural liquid agent, our previous exprience have found autologous blood to be quite safe and effective for preoperative GGNs localization. Autologous blood is of high histocompatibility and cause no tissue rejection. It is not easy to form thrombosis even if it infiltrates into the pulmonary vessels. Due to its natural immune compatibility, autologous blood have prove its safety in localization even for children patients [5, 6]. Based on these advantages and easy accessibility, autologous blood seemed to be an ideal marking material for nodules localization.

Compared to the complex micro-coil deploying process, autologous blood injection seems to be a more simple and prompt procedure. Since the puncture point, injection depth, and direction were predetermined, the puncture and injection could be performed in a single step. This novel localization technique allows positioning to be more accurate and saves time without any adjustment, which results in shorter average duration for localization procedure and accordingly reduced CT scan radiation to patients. A big concern for autologous blood localization must be its potential dissipation or absorption in lung parenchyma. McDonnell et al. [6] used methylene blue mixed with autologous blood to prevent dissipation. While our technique eliminates the use of methylene blue, alleviating the potential risk of allergy. Furthermore, a distinct and accurate subpleural tatoo could still be inspected without diffusion even 12 h after blood injection in our study. Surgeons could easily confirmed the position of targeted GGNs by inspection of these pleural tatoo due to the relative slow diffusion rate of autologous blood. Fariha Sheikh et al. [5] has reported a high successful localization rate (82%) of nonpleural-based pulmonary nodules by using only autologous blood injection in pediatric patients. Our study showed a relatively higher successful targeting rate, with 32 of 34 (94%) patients underwent a complete resection of the nodule for diagnosis by VATS. We think a individualized injection amount in correspondence to the depth of targeted nodule in our study may contribute to the improvement for nodule identification. Based on the above evidence, this comparative study indicated that CT guided localization by autologous blood injection could be an effective and easy alternative for preoperative GGNs localization.

Regarding complications, a relatively lower complication rate (3.1%) was observed in autologous blood localization group in our study. As we know, pneumothorax was the most common finding after a transpleural needle puncture procedure. No torsion to parenchyma by foreign inplant material allows for a lower incidence of punctural complication and favorable tolerance of patients’ self feeling. Futhermore, as an easily accessible agent with rich fibrinogen, autologous blood can promote coagulation and reduce the risk of pulmonary hemorrhage. These characteristics of autologous blood were reported to be used in treating spontaneous pneumothorax and gastroduodenal ulcer bleeding [21, 22]. Accordingly, lower incidence of punctural pneumothorax and pulmonary hemorrhage was observed in our autologous blood group. Another advantage of autologous blood technique must be mentioned is its unequalled cost advantage: the cost for autologous blood localization method is nearly 1/5 of the cost in micro-coil group, which may be more economical and welcomed especially for a patient with multiple GGNs.

Autologous blood localization does have its own disadvantages: a longer duration is needed to locate the target nodule position during thoracoscopic surgery. The sign of successful localization of autologous blood is the formation of subpleural hematoma. However, pleural adhesion or anthracotic pigmentation would increase the failure rate because the puncture hole or hematoma of the visceral pleura cannot be identified easily. Another problem that must be mentioned is that, autologous blood hematoma is difficult to be confirmed by palpation especially for those small and deep GGNs. So in case of hematoma absorption, our experience indicate the interval between localization procedure and surgical resection should better be within 24 h.

This single institutional study had several limitations, including the retrospective design and the relatively small sample size. Furthermore, the study population consisted only of selected patients with one single GGN requiring localization, the feasibility of this novel localization in patients of multi-GGNs cannot be validated. Finally, there may be other important variables (pain score or allergy reaction rate) not included in our database, thus may leading to some bias of our results. Nonetheless, to the best of our knowledge, this study is the first comparative study to evaluate applicability of autologous blood in pulmonary nodule localization. A larger sample randomized and prospective study is also required to verify the clinical application value of this localization procedure.

## Conclusion

In conclusion, CT-guided autologous blood injection was feasible and safe for GGNs localization followed by VATS resection. CT guided autologous blood localization is an effective and more economical, safe method for preoperative lung nodule localization and associated with fewer complications compared to micro-coil localization.

## Data Availability

The data that support the findings of this study are available from the corresponding author upon reasonable request.

## Abbreviations

CT: Computed tomography
GGNs: Ground-glass nodules
VATS: Video-assisted thoracoscopic surgery
WR: Wedge resection
